# Development of a Delirium Risk Predication Model among ICU Patients in Oman

**DOI:** 10.1155/2022/1449277

**Published:** 2022-07-31

**Authors:** Rasha Khamis Al-Hoodar, Eilean Rathinasamy Lazarus, Omar Alomari, Omar Alzaabi

**Affiliations:** College of Nursing, Sultan Qaboos University, Muscat, Oman

## Abstract

**Background:**

Delirium is a common disorder among patients admitted to intensive care units. Identification of the predicators of delirium is very important to improve the patient's quality of life.

**Methods:**

This study was conducted in a prospective observational design to build a predictive model for delirium among ICU patients in Oman. A sample of 153 adult ICU patients from two main hospitals participated in the study. The Intensive Care Delirium Screening Checklist (ICDSC) was used to assess the participants for delirium twice daily.

**Result:**

The results showed that the incidence of delirium was 26.1%. Multiple logistic regression analysis showed that sepsis (odds ratio (OR) = 9.77; 95% confidence interval (CI) = 1.91–49.92; *P* < 0.006), metabolic acidosis (odds ratio (OR) = 3.45; 95% confidence interval [CI] = 1.18–10.09; *P*=0.024), nasogastric tube use (odds ratio (OR) 9.74; 95% confidence interval (CI) = 3.48–27.30; *P* ≤ 0.001), and APACHEII score (OR = 1.22; 95% CI = 1.09–1.37; *P* ≤ 0.001) were predictors of delirium among ICU patients in Oman (*R*^2^=0.519, adjusted *R*^2^=0.519, *P* ≤ 0.001).

**Conclusion:**

To prevent delirium in Omani hospitals, it is necessary to work on correcting those predictors and identifying other factors that had effects on delirium development. Designing of a prediction model may help on early delirium detection and implementation of preventative measures.

## 1. Introduction

Delirium is defined by the Diagnostic and Statistical Manual of Mental Diseases, Fifth Edition (*DSM-5*) as an acute and fluctuating disturbance in attention or awareness that is accompanied by a change in baseline cognition [1]. Delirium has been reported to occur in 9% [2] to 54.9% [3] of patients admitted to intensive care units. Alarmingly, approximately 80% of patients treated in ICUs manifested delirium [4].

ICU delirium is a serious disorder, which has been associated with prolonged mechanical ventilation use [5], longer ICU length of stay [6], hospital stay [7], a 3-fold increase in 6-month mortality rate [8], and increased healthcare costs [9]. However, worsening functional and cognition status after ICU discharge were associated with delirium [10]. Several predictors identified from literature that precipitate delirium development. Mechanical ventilator use was found to be one of the factors that can predict developing delirium [11]. Another factor was physical restraint use concluded that patients on physical restraints were more likely to develop delirium compared to patients without physical restraints [12]. In addition to that, use of sedative drugs was linked to delirium and the study indicated that the patients who received any sedative drugs had 2.61 times the risk for developing delirium compared to patients who did not receive any sedative drug during their stay in ICU [13].

A rise in the chronic disease worldwide [14] is contributing to rise the incidence of delirium, as older adults are more vulnerable group for delirium especially who are hospitalized due to severely compromised conditions [15]. Therefore, a large number of people are at high risk for developing delirium when admitted to hospitals especially in a critical care unit. Several predictive models for intensive care patients had been developed [16–18]. Up to researcher knowledge, there were no studies that explored the predictors of delirium among ICU patients in Oman and there is no evidence-based prediction model for general intensive care patients available. Identifying predictive risk factors in this study will help healthcare professionals in Oman develop a prediction model to help them predict which patients are at higher risk of developing delirium. This model would facilitate creating a revised policy on delirium reduction and introducing therapeutic interventions to prevent or mitigate the harmful effects of delirium on ICU patients. Studying the impact of delirium in the term of ICU length of stay and mortality is very critical to raise awareness among clinicians and healthcare policymakers about the effects of this preventable complication. Designing of the prediction model will help the higher administration stakeholders and decision makers in creating a new guideline on delirium prevention and implementing preventive measures to minimize the occurrence of delirium and enhance patient quality of life. Additionally, the financial and expense consequences of delirium occurrence would be minimized.

One of the essential health research priorities of the Ministry of Health in Oman is patient safety [19]. Delirium prevention is foreseen as an integral parameter for patient safety [20], which will help to reduce the incidence, duration of delirium and its severity. So, this study will help to identify the causes of delirium to set preventive measures to enhance the quality of patient care and reduce the economic burden on the healthcare system caused by the epidemic burden of COVID-19. Additionally, preventive techniques can be prioritized because of the need for personnel and on directing the staff's attention on pulmonary management during delirium.

A review of the literature found a dearth of research examining delirium in intensive care unit patients in the Arab area in general and in Oman in particular. Numerous trials have been conducted at the international level to examine delirium of patients admitted to intensive care units. The bulk of prior research has been done in Western countries, which have somewhat different histories than Arab countries. Additionally, no trials have been conducted in Oman to determine the prevalence of delirium and the causes correlated with it, and nothing is known regarding the predictive factors for delirium in ICU patients in Oman. Due to a lack of evidence, it is necessary to determine the prevalence of delirium and the risk factors correlated with it in the Oman community, as well as to examine the outcomes of delirium. The current study used a prospective and retrospective method and a regression model-building technique to determine the predictors of delirium.

## 2. Materials and Methods

### 2.1. Study Design and Purpose

The study used a prospective observational research design to determine the predictors of delirium among ICU patients admitted in Oman and to develop a delirium prediction model for intensive care patients in Oman.

### 2.2. Sample and Setting

There were two major tertiary hospitals were selected to conduct the study. They both belong to government of Sultanate of Oman and located at Muscat, the capital city of Oman. Based on the inclusion criteria, the study recruited 153 patients from all ICUs (Adult, Coronary Care and Post Cardiac Surgery). We have collected the data from September to December 2020 among 153 patients using convenience sampling approach. Using the logistic regression model with 5% significance and 80% power, we calculated the sample size considering the number of independent variables in the study.

### 2.3. Measurements

#### 2.3.1. Intensive Care Delirium Screening Checklist (ICDSC)

The Intensive Care Delirium Screening Checklist includes eight items (altered level of consciousness, inattention, disorientation, hallucination/delusions/psychosis, psychomotor agitation or retardation, inappropriate speech or mood, sleep wake/cycle disturbance, and symptom fluctuation) based on DSM criteria to assess the occurrence of delirium developed by Bergeron et al. [21]. The ICDSC is a very simple and standardized tool that can be handled by any professional without any formal training. The investigators need to observe the symptoms and need not to ask any questions to the patients to answer and the score more than four indicates the presence of delirium [21]. The English version of the Cronbach *α* of the tool is 0.839 with the sensitivity and specificity of 81.0% and 87.7%, respectively [22].

#### 2.3.2. Acute Physiology and Chronic Health Evaluation II (APACHEII)

The APACHEII was designed to measure of disease severity developed by Knaus et al. [23]. The tool consists of 12 routine physiological points, age points, and chronic health points measured within the first 24 hours of ICU admission [23]. The minimum score of 0 and a maximum score of 71 can be obtained by for patients, each item was scored from 0 to 4 (Zero means most normal and 4 means most abnormal) [23]. The sensitivity and the specificity of the tool was of 87.5% and 79.0%, respectively [24].

#### 2.3.3. Sequential Organ Failure Assessment Score(SOFA)

The SOFA scoring tool consists of six systems, measuring the degree of each system failure like cardiac, cardiovascular, hepatic, coagulation, renal, and neurological, ranging from zero to four and the patient may score from zero to 24 as a total, with a higher score suggesting worsening organ dysfunction [25]. The SOFA scoring tool was developed by European Society of Intensive Care Medicine's Working Group on Sepsis-Related Problems in 1994 [25]. For predicting hospital mortality, it had a sensitivity of 85% and specificity of 73.9% for predicting hospital mortality [26].

### 2.4. Data Collection Procedure

The researchers contacted the ICU mangers and gave them an outline of the aim, processes, and significance. Then, a two-weeks of training course in delirium evaluation using the ICDSC began for nurses. Patients admitted in the ICU more than 24 hours, able to understand Arabic or English, above 18 years of age, and transferred from various other hospitals in and around Oman were included in the study. Patients presented with an unresponsive and comatose condition, suffering from alcohol withdrawal syndrome or alcohol-induced delirium, cognitive disorders, COVID 19, admitted in the ICU for less than 24 hours, and patients readmitted to the ICU after participating in the study were not included in the study. Each patients were provided with a package consists of information sheet and a consent form. The researchers reviewed the patients details from the hospital records like admission notes at the ICU, patient's demographic information, past and present medical history including comorbidity and smoking history. Along with that the researcher and the ICU nurses assessed the patient for the presence of delirium every 12 hours using ICDSC scale and calculated patient's SOFA and APACHE II score. Delirium was measured as a binary variable. If a patient had delirium on at least one examination during their ICU stay, they were listed as having delirium.

### 2.5. Ethical Consideration

We obtained ethical approval and permission to conduct from College of Nursing at Sultan Qaboos University (CON/MSN/2020/5), Royal Hospital ethical committee (SRC#50/2020), and College of Medicine at Sultan Qaboos University (SQU-EC/093/2020). The researcher secured permission to use the tool. The researcher taken written informed consent from all the participants and the participants were recruited on voluntary basis. The researcher did not collect any identification data of the patients.

### 2.6. Statistical Analysis

Statistical Package for Social Science (SPSS) Software program version 23 was used to analyze all data. Univariate tests were used to screen potential predisposing and precipitating predictors of delirium. For categorical variables, *χ*^2^ tests were used. A point-biserial correlation was used to compare continuous variables. Binary logistic regression analysis was used to assess the independent effect of different factors on the development of delirium.

## 3. Results

In this study, 153 patients were screened for delirium and the study. Out of the total, 40 developed delirium (26.1%). Among them, there were 98 men (64.1%) and 55 women (35.9%). The participants were on an average 53 years old (SD 19.6). Around 88 people (57.5%) had medical problems, and the majority of the participants had co-morbidity 165 (92.2%). The majority of them were nonsmokers (83.7%). The rest of the other characteristics of the participants are summarized in Table 1.

**Figure 1:**
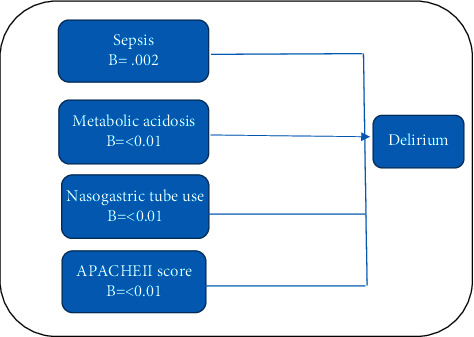
Model of predictors of overall patient safety culture.

Univariate analysis was undertaken to determine the relationship between study variables and delirium. The results showed that sepsis (*P*=0.002), metabolic acidosis (*P* < 0.01), nasogastric tube use (*P* < 0.01), sedation use (*P*=0.017), creatinine level (*P*=0.014), and APACHEII score (*P* < 0.01) were all found to be associated with delirium in univariate analyses. Multiple logistic regression analysis was used to identify the predictors of delirium. The independent variables that were correlatedwith delirium in the bivariate analyses (sepsis, metabolic acidosis, nasogastric tube use, APACHEII score, sedation use, and creatinine level) were included in the initial regression model. Regression analysis showed that sepsis (odds ratio (OR) = 9.77; 95% confidence interval (CI) = 1.91–49.92; *P* < 0.006), metabolic acidosis (odds ratio (OR) = 3.45; 95% confidence interval (CI) = 1.18–10.09; *P*=0.024), nasogastric tube use (odds ratio (OR) 9.74; 95% confidence interval (CI) = 3.48–27.30; *P* ≤ 0.001), and APACHEII score (OR = 1.22; 95% CI = 1.09–1.37; *P* ≤ 0.001) were independently associated with delirium and those were predicted delirium among ICU patients in selected hospitals in Oman (*R*^2^=0.519, adjusted *R*^2^=0.519, *P* ≤ 0.001).  Table 2 details the univariate and multiple logistic regression results.  Figure 1 shows the model of predictors of delirium.

## 4. Discussion

This study was conducted to develop a delirium prediction model for intensive care patients in selected hospitals in Oman. The current study showed that the incidence of delirium among ICU patients was 26.1%. In this study, the delirium prediction model among ICU patients was developed. It is the first delirium predication study for ICU patients in Oman. The result of the current study showed that sepsis is significant predictor of delirium. This is in line with study investigated the predicators of delirium and had same conclusion [27, 28]. Sepsis triggers a systemic inflammatory response and the release of cytokines and/or bacterial metabolites, which can disturb the blood-brain barrier, resulting in hypoxia, physiological changes in the brain, and insufficient cerebral perfusion, resulting in delirium [29].

Moreover, current study result was consistent with current study findings that showed metabolic acidosis associated with delirium development and predicted delirium development [30]. Reduction in acetylcholine activity in the brain due to electrolyte imbalance may precipitate delirium development [31]. Other studies showed that significant association between severity of the illness and delirium [28, 32]. Those results were consistent with current study findings. The results of the current study revealed that patients with a nasogastric tube are more likely to experience delirium, which is consistent with previous study [33]. This might be because the presence of nasogastric tube is indicated for severity of the disease which is significant risk factor for delirium development. Therefore, there is a need to assess for these lines on a regular basis in order to facilitate early removal.

The findings of this study have implications for nursing and health policymakers, including mangers, nursing administrators, and policymakers, who should be aware about the predictors of delirium and therefore plan for strategies to treat and prevent delirium through targeting high risk patients for delirium. In addition, the results may encourage the health care professionals to screen any admitted patients for the presence of any predictors of delirium and to take the necessary measures to prevent delirium development and incorporate multidisciplinary team involvement to manage the case.

The results of this study can also be used as a baseline and source of data for future research studies. Further research is recommended to validate the predication model and compare it with the previous prediction models. This study has some limitation, it used a convenience sampling and had a small sample size in the delirium group, which may have affected the statistical analysis and restricted the generalizability of the findings.

## 5. Conclusion

In conclusion, delirium among ICU patients is common and is linked to multiple negative outcomes. For example, increased ICU length of stay, hospital length of stay, and prolonged mechanical ventilation use in the ICU. Identification of predicators of delirium is an essential component for successful early detection and management of delirium through planning for effective preventative strategies by top management in any healthcare institutions. Delirium prevention is one of the essential parameters to protect and maintain patient's safety and patient safety is stated as a goal for any health organization.

## Figures and Tables

**Table 1:** Main clinical and demographic characteristics of the study participants(*N* = 153).

Variables	Total sample (*n* = 153)
*ICDSC* ^*a*^	
Delirium	40 (26.1)
No-delirium	113 (73.9)

Age (Years)^b^	53 ± 19.6 (18–78)

*Gender* ^*a*^	
Male	98 (64.1)
Female	55 (35.9)

*Diagnosis* ^*a*^	
Medical	88 (57.5)
Surgical	65 (42.5)

*Emergency surgery or trauma* ^*a*^	
No	135 (88.2)
Yes	18 (11.8)

*Comorbidity* ^*a*^	
None	12 (7.8)
One diseases	39 (25.5)
Two diseases	30 (19.6)
Three diseases	71 (46.4)
Six diseases	1 (0.7)

*Sepsis* ^*a*^	
No	140 (91.5)
Yes	13 (8.5)

*Vasopressor use* ^*a*^	
No drug	57 (37.3)
One drug	79 (51.6)
Three drug	11 (7.2)
Four drugs	6 (3.9)

*Ventilator use* ^*a*^	
No	44 (28.8)
Yes	109 (71.2)

*Sedation* ^*a*^	
No drug	50 (32.7)
One drug	45 (29.4)
Two drugs	58 (37.9)

*Metabolic acidosis* ^*a*^	
No	88 (57.5)
Yes	65 (42.5)

*Bladder catheter use* ^*a*^	
No	20 (13.2)
Yes	133 (86.9)

*Nasogastric tube use* ^*a*^	
No	104 (68)
Yes	49 (32)

APACHEII (points)^b^	18 ± 5.6 (7–28)
SOFA (points)^b^	8 ± 3.2 (0–14)
SAPS II (points)^b^	48 ± 14.7 (22–85)
Bilirubin (mmol/L)^b^	20 ± 20 (3–86)
Creatinine (mmol/L)^b^	159 ± 158 (37–872)
Sodium (mEq/L)^b^	137 ± 8 (117–159)
ICU length of stay (days)^b^	6 ± 8 (2–51)

^a^ Number (percentage), ^b^Mean ± standard deviation (range).

**Table 2:** Results of the univariate and multiple logistic regression analyses.

Variables	Univariate analysis, *P* value	Multiple regression analysis		
		*P* value	Standardized coefficients beta	Odds ratio (95% confidence interval)
Sepsis	0.002	0.006	2.279	9.77 (1.91–49.92)
Metabolic acidosis	<0.01	0.024	1.238	3.45 (1.18–10.09)
Nasogastric tube use	<0.01	0.000	2.277	9.74 (3.48–27.30)
APACHEII score	<0.01	0.001	0.200	1.22 (1.09–1.37)
Sedation use	0.017	0.799	0.087	1.09 (0.56–2.12)
Creatinine level	0.014	0.189	−0.003	1.00 (0.99–1.00)

Dependent Variable—ICU delirium.

## Data Availability

The data used to support the findings of this study are included within the article.

## References

[1] American Psychatric Association (2013). *Diagnostic and Statistical Manual of Mental Disorders (DSM-5®)*.

[2] Abla H.-A., Alshraideh J. A. (2019). Delirium post‐cardiac surgery: incidence and associated factors. *Nursing in Critical Care*.

[3] Kotfis K., Szylińska A., Listewnik M. (2018). Early delirium after cardiac surgery: an analysis of incidence and risk factors in elderly (a≥65 years) and very elderly (a≥80 years) patients. *Clinical Interventions in Aging*.

[4] Hayhurst C. J., Pandharipande P. P., Hughes C. G. (2016). Intensive care unit delirium: a review of diagnosis, prevention, and treatment. *Anesthesiology*.

[5] Tilouche N., Hassen M. F., Ali H. B. S., Jaoued O., Gharbi R., El Atrous S. S. (2018). Delirium in the intensive care unit: incidence, risk factors, and impact on outcome. *Indian Journal of Critical Care Medicine*.

[6] Sharma A., Malhotra S., Grover S., Jindal S. K. (2012). Incidence, prevalence, risk factor and outcome of delirium in intensive care unit: a study from India. *General Hospital Psychiatry*.

[7] Yamaguchi T., Tsukioka E., Kishi Y. (2014). Outcomes after delirium in a Japanese intensive care unit. *General Hospital Psychiatry*.

[8] Schubert M., Schurch R., Boettger S. (2018). A hospital-wide evaluation of delirium prevalence and outcomes in acute care patients-a cohort study. *BMC Health Services Research*.

[9] Vasilevskis E. E., Chandrasekhar R., Holtze C. H. (2018). The cost of ICU delirium and coma in the intensive care unit patient. *Medical Care*.

[10] Patel M. B., Bednarik J., Lee P. (2018). Delirium monitoring in neurocritically ill patients: a systematic review. *Critical Care Medicine*.

[11] Tsuruta R., Nakahara T., Miyauchi T. (2010). Prevalence and associated factors for delirium in critically ill patients at a Japanese intensive care unit. *General Hospital Psychiatry*.

[12] Mori S., Takeda J. R. T., Carrara F. S. A., Cohrs C. R., Zanei S. S. V., Whitaker I. Y. (2016). Incidence and factors related to delirium in an intensive care unit. *Revista da Escola de Enfermagem da USP*.

[13] Rasheed A. M., Amirah M., Abdallah M., Awajeh A. M., Parameaswari P. J., Al Harthy A. (2019). Delirium incidence and risk factors in adult critically ill patients in Saudi Arabia. *Journal of Emergencies, Trauma, and Shock*.

[14] Andersen K., Gudnason V. (2012). Chronic non-communicable diseases: a global epidemic of the 21st century. *Laeknabladid*.

[15] Mattoo S. K., Grover S., Gupta N. (2010). Delirium in general practice. *Indian Journal of Medical Research*.

[16] Chen Y., Du H., Wei B.-h., Chang X.-n., Dong C.-m. (2017). Development and validation of risk-stratification delirium prediction model for critically ill patients: a prospective, observational, single-center study. *Medicine*.

[17] Boogaard M. V. D., Pickkers P., Slooter A. J. C. (2012). Development and validation of PRE-DELIRIC (PREdiction of DELIRium in ICu patients) delirium prediction model for intensive care patients: observational multicentre study. *BMJ*.

[18] Wassenaar A., van den Boogaard M., van Achterberg T. (2015). Multinational development and validation of an early prediction model for delirium in ICU patients. *Intensive Care Medicine*.

[19] MOH (2014). Health Research Priorities. https://mohcsr.gov.om/wp-content/uploads/2015/03/research-priority-8.4.2014.pdf.

[20] Reston J. T., Schoelles K. M. (2013). In-facility delirium prevention programs as a patient safety strategy: a systematic review. *Annals of Internal Medicine*.

[21] Bergeron N., Dubois M. J., Dumont M., Dial S., Skrobik Y. (2001). Intensive care delirium screening checklist: evaluation of a new screening tool. *Intensive Care Medicine*.

[22] Detroyer E., Timmermans A., Segers D. (2020). Psychometric properties of the intensive care delirium screening checklist when used by bedside nurses in clinical practice: a prospective descriptive study. *BMC Nursing*.

[23] Knaus W. A., Draper E. A., Wagner D. P., Zimmerman J. E. (1985). Apache II: a severity of disease classification system. *Critical Care Medicine*.

[24] Munyua B. K. (2018). Evaluating the validity of apache li as a predictor of ICU mortality for the critically III patients at Knh’scritical care units.

[25] Vincent J.-L., Moreno R., Takala J. (1996). The SOFA (Sepsis-related Organ Failure Assessment) score to describe organ dysfunction/failure. *Intensive Care Medicine*.

[26] Laimoud M., Alanazi M. (2020). The validity of sofa score to predict mortality in adult patients with cardiogenic shock on venoarterial extracorporeal membrane oxygenation. *Critical Care Research and Practice*.

[27] Duceppe M.-A., Williamson D. R., Elliott A. (2019). Modifiable risk factors for delirium in critically ill trauma patients: a multicenter prospective study. *Journal of Intensive Care Medicine*.

[28] Lahariya S., Grover S., Bagga S., Sharma A. (2014). Delirium in patients admitted to a cardiac intensive care unit with cardiac emergencies in a developing country: incidence, prevalence, risk factor and outcome. *General Hospital Psychiatry*.

[29] Tsuruta R., Oda Y. (2016). A clinical perspective of sepsis-associated delirium. *Journal of intensive care*.

[30] Wu C., Zhu Y., Li G. (2020). Incidence and risk factors of delirium in ICU patients. *Journal of Third Military Medlical University*.

[31] Aldemir M., Özen S., Kara I. H., Sir A., Baç B. (2001). Predisposing factors for delirium in the surgical intensive care unit. *Critical Care*.

[32] Shi C.-M., Wang D.-X., Chen K.-S., Gu X.-E. (2010). Incidence and risk factors of delirium in critically ill patients after non-cardiac surgery. *Chinese Medical Journal*.

[33] Xing J., Yuan Z., Jie Y., Liu Y., Wang M., Sun Y. (2019). Risk factors for delirium: are therapeutic interventions part of it?. *Neuropsychiatric Disease and Treatment*.

